# Expression of testicular genes in haematological malignancies

**DOI:** 10.1038/sj.bjc.6690824

**Published:** 1999-12

**Authors:** S H Lim, S Austin, E Owen-Jones, L Robinson

**Affiliations:** Department of Haematology, University of Wales College of Medicine, Cardiff, UK

**Keywords:** CT antigens, leukaemia, myeloma

## Abstract

The gene expression of a new group of tumour antigens known as cancer/testis (CT) antigens is now well-recognized in some solid tumours. However, their expression in haematological malignancies remained unclear. In this study, we have used reverse transcription polymerase chain reaction and Southern blot analysis to examine the presence of transcripts for the three CT antigens, NY-ESO-1, SSX2 and SCP1 in haematological malignant cells. We found that transcripts for SCP1 could be detected in 10% of myeloma, 5.7% of acute myeloid leukaemia and 23% of chronic myeloid leukaemia. In contrast, NY-ESO-1 and SSX2 were not detected in any of the 107 tumour samples. © 1999 Cancer Research Campaign


*British Journal of Cancer* (1999) **81**(7), 1162–1164
© 1999 Cancer Research Campaign
Article no. bjoc.1999.0824

### Expression of testicular genes in haematological malignancies

**SH Lim, S Austin, E Owen-Jones and L Robinson**

Department of Haematology, University of Wales College of Medicine, Cardiff, UK

**Summary** The gene expression of a new group of tumour antigens known as cancer/testis (CT) antigens is now well-recognized in some solid tumours. However, their expression in haematological malignancies remained unclear. In this study, we have used reverse transcription polymerase chain reaction and Southern blot analysis to examine the presence of transcripts for the three CT antigens, NY-ESO-1, SSX2 and SCP1 in haematological malignant cells. We found that transcripts for SCP1 could be detected in 10% of myeloma, 5.7% of acute myeloid leukaemia and 23% of chronic myeloid leukaemia. In contrast, NY-ESO-1 and SSX2 were not detected in any of the 107 tumour samples. © 1999 Cancer Research Campaign

**Keywords:** CT antigens; leukaemia; myeloma

Various laboratory studies have indicated that haematological malignancies may be amenable to immune targeting. In B-lymphoma (Kwak et al, 1992; Wen and Lim, 1997) and multiple myeloma (Wen et al, 1998*a*, 1998*b*; Lim and Bailey-Wood, 1999), efforts have been concentrated primarily on the targeting of the idiotypic proteins or peptides. In leukaemias, various fusion genes arising due to specific chromosomal translocation have been the targets (Pawelec et al, 1996). Whole cell immunization strategies (Choudhury et al, 1997; Coleman et al, 1997; Lim et al, 1998) further supported the presence of immunogenic peptides, which could be recognized by host T-cells. A recent study (Sahin et al, 1995) suggested that tumour cells often elicit multiple specific immune responses in the autologous host. Whilst some tumour antigens associated with haematological malignancies are well known, others remain to be determined.

A new class of tumour antigens, known as CT (cancer/testis) antigens, has recently been described (Old and Chen, 1998). They include members of the *MAGE* family, *BAGE* and *GAGE*. They are normal testicular antigens expressed aberrantly in tumour cells. The restricted normal expression of these antigens makes them ideal molecules for immune targeting. Most of these antigens have been isolated through serological screening of expression recombinant libraries constructed from tumour RNA. Whilst the expression of these antigens in normal tissues and in solid tumour cells are established (Chen et al, 1997), evidence relating to the pattern of expression in haematological malignancies is scarce. Expression of *MAGE-1*, *MAGE-3*, *BAGE* and *GAGE-1,2* have been investigated in leukaemias (reviewed by van den Eynde and van der Bruggen, 1997) but their expression was not demonstrated except in T-cell leukaemias related to HTLV-1 infection. In this study, we have examined the expression of a panel of three known CT antigens, SSX2, NY-ESO-1 and SCP1, in tumour cells derived from a cohort of patients with haematological malignancies and leukaemia cell lines, CEM, HL60 and Molt 4.

*Received 9 December 1998*
*Revised 27 April 1999*
*Accepted 29 April 1999*

*Correspondence to:* SH Lim, Myeloma and Transplantation Research Center, University of Arkansas for Medical Science, 4301 West Markham, Slot 776, Little Rock, Arkansas 72205, USA

### MATERIALS AND METHODS

A total of three leukaemia cell lines (CEM, HL60 and Molt 4), six normal bone marrow and 107 tumour specimens (30 chronic myeloid leukaemia (CML), 52 acute myeloid leukaemia (AML), 20 multiple myeloma (MM) and five acute lymphoblastic leukaemia (ALL) were analysed. All samples were collected at disease presentation without any prior therapy. All 30 CML were in chronic phase of the disease. The French–American–British (FAB) subtypes of the 52 AML were: ten $M_1$, 26 $M_2$, four $M_3$, nine $M_4$ and three $M_5$.

Total RNA was isolated from tumour cells using standard RNAzol method. The mRNA expression of these CT antigens was evaluated using reverse transcription (RT) and polymerase chain reaction (PCR). RT was carried out in each sample using random hexamer primers on 1 μg of total RNA. The PCR primers for each of the CT antigen are: NY-ESO-1: ESO-1A, 5′-CAC ACA GGA TCC ATG GAT GCT GCA GAT GCG G-3′ and ESO-1B, 5′-CAC ACA AAG CTT GGC TTA GCG CCT CTG CCC TG-3′ (Chen et al, 1997); SSX2: SSX2A, 5′-GTG CTC AAA TAC CAG AGA AGA TC-3′ and SSX2B, 5′-TTT TGG GTC CAG ATC TCT CGT G-3′ (Gure et al, 1997); SCP1: SCP1A, 5′-CTA CTG TCT GCA GCT TGG-3′ and SCP1B, 5′-CTC GTT TCG AGC TCA GTT-3′. These primer pairs amplify cDNA segments of 300–400 bp in lengths. PCR was performed using 35 amplification cycles in a thermal cycler at annealing temperature of 60°C for NY-ESO-1, 65°C for SSX2 and 55°C for SCP1. Each set of PCR reactions contained a positive control of cDNA derived from normal testis and a negative control of all the PCR reaction mixture except for cDNA which was substituted with water. The integrity of RNA was checked in each PCR reaction by multiplex amplification for the c-*abl* gene segment. All positive amplifications, as visualized on ethidium bromide agarose gels, were confirmed by two independent RT-PCR and the specificity of the PCR products by Southern blot analysis using the respective probes derived from the PCR amplification products of testicular cDNA.

**Table 1** Frequency of mRNA expression

| | SSX2 | NY-ESO-1 | SCP1 |
|---|---|---|---|
| Normal bone marrow | 0/6 | 0/6 | 0/6 |
| CML | 0/30 | 0/30 | 7/30 |
| AML | 0/52 | 0/52 | 3/52 |
| MM | 0/20 | 0/20 | 2/20 |
| ALL | 0/5 | 0/5 | 0/5 |
| Cell lines | | | |
| CEM | ND | ND | ND |
| HL60 | ND | ND | ND |
| Molt 4 | ND | ND | ND |

CML = chronic myeloid leukaemia; AML = acute myeloid leukaemia; MM = multiple myeloma; ALL = acute lymphoblastic leukaemia; ND = not detected.

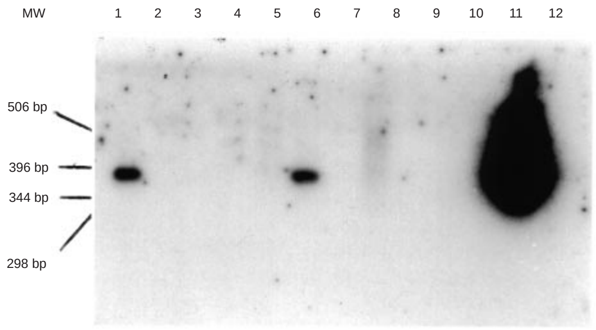

**Figure 1** RT-PCR analysis of mRNA for SCP1. Following gel electrophoresis, DNA was transferred overnight onto a nitrocellulose membrane. The membrane was then hybridized overnight at 60°C to a [$\gamma^{32}$P]-ATP end-labelled probe derived from SCP1 amplification of normal testicular cDNA. Washing was performed under high stringency conditions, with the final wash of the filter at 0.2 × standard saline citrate and 0.1% sodium dodecyl sulphate at 60°C for 20 min. DNA was visualized by autoradiography after overnight exposure. Lanes 1 and 6 showed positive signals of the expected size from the PCR amplification of tumour cell cDNA from two CML patients (lanes 2, 3, 4 and 8 = AML; lane 5 = ALL; lanes 7 and 9 = MM; lane 10 = CML). In contrast, the signal in lane 11, derived from amplification of normal testicular cDNA, was much stronger. Lane 12 consisted of a negative control amplification

### RESULTS

Table 1 shows the mRNA expression of these CT antigens in tumour cells derived from haematological malignancies. All three antigens were not detected in normal bone marrow. SCP1 mRNA was detected in 2/20 (10%) myeloma, 3/52 (5.7%) AML and 7/30 (23%) CML samples. Of the three AML giving a PCR product, SCP1 was detected in two $M_2$ and one $M_4$. It was, however, not detected in all three leukaemia cell lines. In all cases, amplification of *c-abl* was successful. Although the PCR was not designed primarily to give accurate mRNA quantitation, the reproducibly much less intense radiography signals obtained from Southern blot analysis when compared to those obtained with normal testicular PCR products would suggest low levels of SCP1 expression in tumour cells derived from haematological malignancies (Figure 1). In contrast to SCP1, SSX2 and NY-ESO-1 were not detected in any of the 107 tumour specimens and three leukaemia cell lines evaluated.

### DISCUSSION

Recent advances in tumour immunology and immunotherapy have been in part due to the successful identification of tumour antigens. In this study, we set out to determine the gene expression of three known CT antigens in haematological malignancies because information relating to these antigens had been derived primarily from solid tumours. Detection of these antigens in haematological malignancies may permit their use for immune targeting in these malignancies.

Using the techniques of RT-PCR followed by Southern blot analysis, we did not detect any transcripts for NY-ESO-1 and SSX-2 in any of the tumour samples. Taken together with a previous study showing the lack of expression of NY-ESO-1 in ten lymphoma samples (Chen et al, 1997) and SSX2 in 14 leukaemia/lymphoma (Tureci et al, 1998*a*), it is likely that these transcripts are rarely expressed in haematological malignancies. In contrast to NY-ESO-1 and SSX-2 and a previous study including 14 leukaemia/lymphoma in which the SCP1 transcript could not be detected (Tureci et al, 1998*b*), we have found that the expression of SCP1 in the tumour cells from some haematological malignancies. SCP1 is involved in the pairing of chromosomes during meiosis. It is at present the only CT antigen with a known function. It has been identified as a member of the CT antigen following serological screening of a recombinant cDNA library derived from tumour cells, indicating its ability to elicit immune responses in the cancer host. Its expression in haematological malignancies has never been previously reported. Although its mRNA expression is probably much lower than that in normal testis, its prevalence, especially amongst patients with CML may suggest a potential role for its use for immune targeting of the leukaemia. This obviously would require the appropriate processing and presentation of the peptide in association with the host MHC molecules. Its low level of expression may not be a problem if primary immune responses could first be triggered with synthetic peptides since target recognition by T-cells requires only very low copy number of peptides on the cell surface. Further work is ongoing to determine the in vivo immunogenicity of the SCP1 antigens in patients with tumour cells expressing the SCP1 mRNA.

### ACKNOWLEDGEMENTS

Funded by the Leukaemia Research Fund, UK, Leukaemia Research Appeal for Wales and the Welsh Bone Marrow Transplant Research Fund.

### REFERENCES

Chen Y-T, Scanlan MJ, Sahin U, Tureci O, Gure A, Tsang S, Williamson B, Stockert E, Pfreundschuh M and Old LJ (1997) A testicular antigen aberrantly expressed in human cancers detected by autologous antibody screeening. *Proc Natl Acad Sci USA* **94**: 1914–1918

Choudhury A, Gajewski JL, Liang JC, Popat U, Claxton DF, Kliche KO, Andreef M and Champlin RE (1997) Use of leukemic dendritic cells for the generation of antileukemic cellular cytotoxicity against Philadelphia chromosome-positive chronic myelogenous leukemia. *Blood* **89**: 1133–1142

Coleman S, Throp D, Fisher J, Bailey-Wood R and Lim SH (1997) Cytokine enhancement of immunogenicity in chronic myeloid leukaemia. *Leukemia* **11**: 2055–2059

Gure AO, Tureci O, Sahin U, Tsang S, Scanlan MJ, Jager E, Knuth A, Pfreundschuh M, Old LJ and Chen Y-T (1997) SSX: A multigene family with several members transcribed in normal testis and human cancer. *Int J Cancer* **72**: 965–971

Kwak LW, Campbell MJ, Czerwinski DK, Hart S, Miller RA and Levy R (1992) Induction of immune responses in patients with B-cell lymphoma against the

© 1999 Cancer Research Campaign surface-immunoglobulin idiotype expressed by their tumors. *N Engl J Med* **327**: 1209–1215
Lim SH and Bailey-Wood R (1999) Idiotypic protein-pulsed dendritic cell vaccination in multiple myeloma. *Int J Cancer* (*in press*)
Lim SH, Coleman S and Bailey-Wood R (1998) In vitro cytokine-primed leukaemia cells induce in vivo T-cell responsiveness in chronic myeloid leukaemia. *Bone Marrow Transplantation* **22**: 1185–1190
Old LJ and Chen Y-T (1998) New paths in human cancer serology. *J Exp Med* **187**: 1163–1167
Pawelec G, Max H, Halder T, Bruserud Ø, Merl A, da Silva P and Kalbacher H (1996) BCR-ABL leukemia oncogene fusion peptides selectively bind to certain HLA-DR alleles and can be recognised by T cells found at low frequency in the repertoire of normal donors. *Blood* **88**: 2118–2124
Sahin U, Tureci O, Schmitt H, Cochlovius B, Johannes T, Schmits R, Stenner F, Luo G, Schobert I and Pfreundschuh M (1995) Human neoplasms elicit multiple specific immune responses in the autologous host. *Proc Natl Acad Sci USA* **92**: 11810–11813
Tureci O, Chen YT, Sahin U, Gure AO, Zwick C, Villena C, Tsang S, Seitz G, Old LJ and Pfreundschuh M (1998*a*) Expression of SSX genes in human tumors. *Int J Cancer* **77**: 19–23
Tureci O, Sahin U, Zwick C, Koslowski M, Seitz G and Pfreundschuh M (1998*b*) Identification of a meiosis-specific protein as a member of the class of cancer/testis antigens. *Proc Natl Acad Sci USA* **95**: 5211–5216
Van den Eynde and van der Bruggen (1997) T cell defined tumor antigens. *Curr Opin Immunol* **9**: 684–693
Wen YJ and Lim SH (1997) T cells recognise the VH complementarity-determining region 3 of the idiotypic protein of B-cell non-Hodgkin's lymphoma. *Eur J Immunol* **27**: 1043–1047
Wen YJ, Ling M, Bailey-Wood R and Lim SH (1998*a*) Idiotypic protein-pulsed adherent peripheral blood mononuclear cell-derived dendritic cells prime immune system in multiple myeloma. *Clin Cancer Res* **4**: 957–962
Wen YJ, Ling M and Lim SH (1998*b*) Immunogenicity and cross-reactivity with idiotypic IgA of VH CDR3 peptide in multiple myeloma. *Br J Haematol* **100**: 464–468

*© 1999 Cancer Research Campaign*
